# The down-regulation of GNAO1 and its promoting role in hepatocellular carcinoma

**DOI:** 10.1042/BSR20130001

**Published:** 2013-09-17

**Authors:** Xiaoyu Pei, Jun Zhang, Lijun Wu, Bin Lü, Xiaojiao Zhang, Dongqin Yang, Jie Liu

**Affiliations:** *Department of Digestive Diseases, Huashan Hospital, Fudan University, Shanghai, China; †Institutes of Biomedical Sciences and Department of Immunology of Shanghai Medical School, Fudan University, Shanghai, China

**Keywords:** cell proliferation, GNAO1, hepatocellular carcinoma (HCC), senescence, CCK-8, cell counting kit-8, DMEM, Dulbecco's modified Eagle's medium, GAPDH, glyceraldehyde-3-phosphate dehydrogenase, GNAO1, guanine nucleotide-binding protein, α-activating activity polypeptide O, HCC, hepatocellular carcinoma, IHC, immunohistochemistry, NC, negative control, NTC, non-transfected control, qPCR, quantitative PCR, RT, reverse transcription, siRNA, small-interfering RNA, SA-β-gal, senescence-associated β-galactosidase

## Abstract

GNAO1 (guanine nucleotide-binding protein, α-activating activity polypeptide O) is a member of the subunit family of Gα proteins, which are molecular switchers controlling signal transductions and whose deregulation can promote oncogenesis. HCC (hepatocellular carcinoma) is one of the malignant tumours around the world, which summons novel biomarkers or targets for effective diagnosis and treatments. The present study was aimed to investigate the expression of GNAO1 in HCC patient tissues and the possible mechanisms by which it took effects. The expression of GNAO1 was detected by IHC (immunohistochemistry) and real-time qPCR (quantitative PCR). Cell proliferation test and cell senescence test were then performed to explore the role of GNAO1 in the occurrence and development of HCC. It was revealed that the level of GNAO1 was comparably less in HCC tissues than in the adjacent tissues. Furthermore, down-regulation of GNAO1 increased cell proliferation, while suppressing the senescence of HCC cells. In conclusion, our findings revealed and confirmed the importance of GNAO1 in HCC, indicating that GNAO1 is a potential biomarker as well as a promising therapeutic target for HCC.

## INTRODUCTION

HCC (hepatocellular carcinoma) is one of the common malignant tumours around the world, and its global incidence rate has already exceeded 1 million per year [1]. China has always been the high incidence area of HCC worldwide, which is about 10 times more than the U.S. and Europe [1]. Because of its high malignancy and rapid progression, diagnoses are frequently made at an advanced stage. Nonetheless, there has been no effective treatment programme yet. Cytotoxic drugs usually show poor therapeutic effect. Therefore, it is particularly important to study the HCC-causing genes and pathogenic mechanism so as to improve the multi-stage HCC diagnosis, drug development and clinical treatment.

GNAO1 (guanine nucleotide-binding protein, α-activating activity polypeptide O; G-α-o, gene name) [2–4] is a member of the subunit family of Gα proteins, which are molecular switchers controlling signal transductions and whose deregulation can promote oncogenesis [5]. GNAO1 was reported to be highly expressed in the cerebrum and enriched obviously in the growth cones of neuronal cells [6]. It was also found to be differentially expressed in the DLPFC (dorsolateral prefrontal cortex) of human cocaine-dependent subjects [7].

Except in neuropsychiatry, the molecular and cellular role of GNAO1 has not been thoroughly investigated. As an active constituent of G-protein, G-α-o is widely believed to couple with, and mediates the physiological effects of, a variety of neuronal receptors, such as GABA (γ-aminobutyric acid) receptor B, dopamine D2 receptors and somatostatin receptors [8]. Activated G-α-o was able to alter the activity of an intracellular second messenger system cascade composed of AC (adenylate cyclase), cAMP and PKA (cAMP-dependent protein kinase) [9–12]. Its expression was also significantly down-regulated and is responsible for the regulation of myocardial intracellular calcium [13].

As for disease association, except neuropsychopathy, GNAO1 has not been thoroughly investigated, either. It was found to be significantly reduced in schizophrenia [8]. Its association with neuroglioma has also been revealed [14].

The functional role of mutant GNAO1 in oncogenesis was studied by Kan, et al., who indicated that GNAO1 might be a tumour suppressor gene [15]. Recently, differential gene expression pattern in HCC was evaluated. Jia, et al. who performed an integrated CNA (chromosomal copy number alteration) analysis and gene expression data, and found that GNAO1 may play an important role in the pathogenesis of HCC [16]. According to these reports, we hypothesized that GNAO1 is probably a novel tumour suppressor gene in HCC. Consequently, to verify our hypothesis, we utilized IHC (immunohistochemistry) and real-time qPCR (quantitative PCR) analysis of tissue samples in this study. Furthermore, we explored and evaluated the role of GNAO1 in the occurrence and development of HCC in cell model, including cell proliferation and senescence [17], hoping to explain its evident relation to HCC.

## MATERIALS AND METHODS

### Frozen tissues and tissue slides

The frozen tissues and the formalin-fixed and paraffin-embedded tissue sections (patient specimens) of HCC patients were obtained from archived files in our department according to the protocol approved by the Ethic Review Committee of Huashan Hospital. 20 pairs of cancerous and paired adjacent non-cancerous tissues from each patient were enrolled.

### RNA extraction, RT (reverse transcription) and real-time qPCR

Total RNA was isolated from frozen tissues by TRI Reagent (Sigma-Aldrich) according to the manufacturer's instructions. Synthesis of cDNA was conducted with 500 ng of total RNA using high capacity cDNA Reverse Transcription Kit (Applied Biosystems). It was initiated by pretreatment at 25°C for 10 min, followed by incubation at 37°C for 120 min and terminated by heating at 85°C for 5 min. Real-time qPCR (10 μl reaction volume) was carried out using SYBR^R^ Premix Ex Taq (Perfect Real Time, Takara) according to the manufacturer's protocol in an Applied Biosystems 7500 sequence detection system. GAPDH (glyceraldehyde-3-phosphate dehydrogenase) was used as internal reference for the normalization of the results. The primer sequences were as follows: 5′-GCACCATTGTGAAGCAGATG-3′ (GNAO1 forward), 5′-ACCATATTCGATGCCCAAAG-3′ (GNAO1 reverse), 5′-GAAGGTGAAGGTCGGAGTC-3′ (GAPDH forward) and 5′-GAAGATGGTGATGGGATTTC-3′ (GAPDH reverse).

### IHC analysis of GNAO1 in fixed tissue sections

The polyclonal rabbit anti-human GNAO1 antibody was purchased from Proteintech. Secondary antibody staining kit was purchased from Dako. The tissue sections were deparaffinized twice in xylene for 10 min each, successively washed with 100%, 95%, 85% and 70% ethanol, and then rinsed in distilled water. To retrieve antigens, tissue sections were incubated in 3% H_2_O_2_ for 20 min. After washing in distilled water three times, tissue sections were incubated in sodium citrate buffer (pH 6.0; preheated to 95°C for 5 min) at 95°C for 15 min. After cooling down to room temperature, they were washed with PBS solution three times for 5 min each.

Tissue sections were probed with GNAO1 antibody (1:100 dilution) for 1–2 h. After two washes with PBS, the tissue sections were incubated with secondary antibody for 1 h and subsequently treated with DAB (diaminobenzidine) peroxidase substrate (Dako) solution for colour development.

Counterstaining of cell nuclei in tissue sections for morphological confirmation was performed with the haematoxylin solution for 1 min and then rinsed with distilled water. After sealed with natural gum, the sections were air dried and examined under a microscope.

### Cell culture and the siRNAs (small interfering RNAs)

Human liver cancer cell line QGY-7703 and human HCC cell line SMMC-7721 (Cell Resource Center of Shanghai Institutes for Biological Sciences, Chinese Academy of Sciences, Shanghai, China) was used to evaluate the cellular effects of the deficient expression of GNAO1. The siRNA sequences for GNAO1 and NC (negative control) were as follows: 5′-GCAGAUGAAGAUCAUCCAUTT-3′ (siGNAO1-1 sense), 5′-AUGGAUGAUCUUCAUCUGCTT-3′ (siGNAO1-1 anti-sense), 5′-GGGCAUCGAAUAUGGUGAUTT-3′ (siGNAO1-2 sense), 5′-AUCACCAUAUUCGAUGCCCTT-3′ (siGNAO1-2 anti-sense), 5′-UUCUCCGAACGUGUCACGUTT-3′ (NC sense) and 5′-ACGUGACACGUUCGGAGAATT-3′ (NC anti-sense). Cells without transfection were used as NTC (non-transfected control).

### Western blotting to confirm the inhibitory effect of GNAO1 siRNAs

Cell lysates were loaded on a SDS/10%PAGE and transferred onto PVDF membrane. After blocking with 5% non-fat dried skimmed milk, the membranes were incubated in the primary antibody at 4°C overnight. After washing three times by TBST (Tris-buffered saline and Tween 20), the membranes were incubated in HRP (horseradish peroxidase)-conjugated secondary antibody (Cell Signaling Technology) for 2 h at room temperature. Protein bands were visualized using Immobilon Western Chemiluminescent Substrate (Millipore) and detected by chemiluminescence imaging system LAS3000 (Fuji) according to the manufacturer's instructions.

### Cell proliferation test

Twenty-four h before transfection, 3000 cells were plated in each well of a 96-well plate. By adding 0.175 μl Lipofectamine™ 2000 (Invitrogen), 3.5 pmol of siRNA was transfected into the cells according to the manual of Lipofectamine™ 2000. The medium was changed to full DMEM-h (Dulbecco's modified Eagle's medium with high glucose, Invitrogen) after 4 h. After 24, 48, 72 or 96 h, the medium was changed to 100 ul fresh DMEM-h with an addition of 10 ul CCK-8 (cell counting kit-8) reagent (Dojindo). After 4 h of incubation, the data of absorbance value which is in accordance with the cell proliferation rate was measured by a microplate reader (Bioteck). Each experiment was performed three times independently.

### Cell senescence test

Cell senescence was assessed by detecting the SA-β-gal activity at pH 6.0. Briefly, 24 h before transfection, 3×10^4^ cells were plated in each well of a 24-well plate. By adding 0.7 μl Lipofectamine™ 2000, 14 pmol of siRNA was transfected into the cells according to the manual of Lipofectamine™ 2000. The medium was changed to full DMEM-h after 4 h. After 72 h, it was washed with PBS and successively incubated with the fixing and working solution of SA-β-gal (senescence-associated β-galactosidase) staining kit (Sigma-Aldrich Corporation) according to the manufactory's manual. The staining degree was reviewed and photographed under a general optical microscope.

## RESULTS

### The down-regulation of GNAO1 in mRNA and protein levels in cancerous tissues

**Figure 1:**
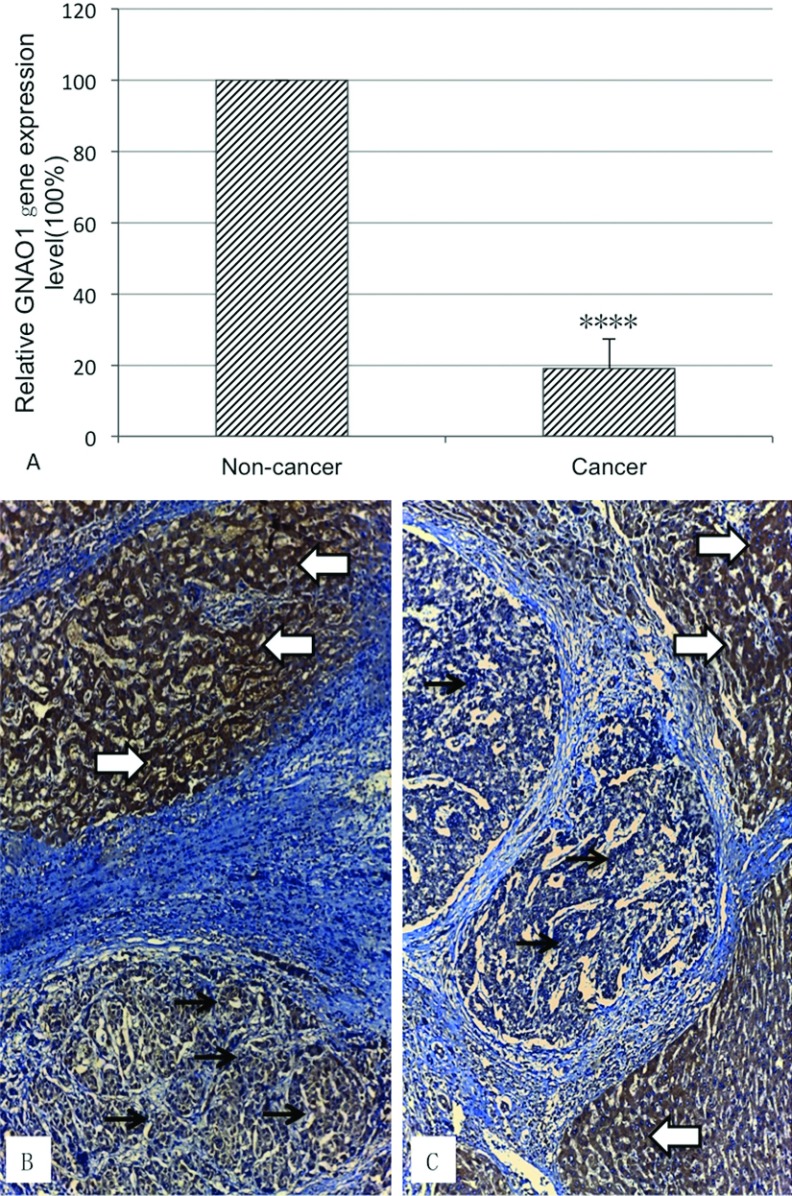
Expression of GNAO1 in HCC and para-tumour tissue Relative expression of GNAO1 gene in frozen cancerous tissues and the adjacent non-cancerous tissues is shown in Panel **A**. mRNA level of GNAO1 gene in cancer was determined with quantitative RT–PCR using human GAPDH as a house-keeping gene control, and expressed in percentage of mRNA level of non-tumour tissue. ‘****’ stands for *P* value less than 0.0001. Representative immunohistochemical images (brown colour) of GNAO1 are shown in liver cancer tissue (**B)** and para-tumoural tissue **(C)**, and are marked with black arrows (cancer cells) and white thick arrows (non-tumoural tissue). The tissues were counter-stained with haematoxylin for nuclei. Magnification: ×200.

Frozen tissues (cancerous and paired adjacent non-cancerous) from a total of 17 subjects were tested for the GNAO1 expression level by RNA isolation, RT and real-time qPCR. Inspiringly, the down-regulation of GNAO1 mRNA transcript was observed in 16 out of 17 frozen cancerous tissues compared with the adjacent non-cancerous tissues of the same subject. The overall down-regulation of GNAO1 mRNA level as mixing the data from all the subjects was also confirmed (Expression fold±S.D=19.04±8.33%), the *p* value was 4.69×10^−6^ (<0.0001) (Figure 1A).

By IHC, we studied and compared the protein levels of GNAO1 in 20 pairs of cancer and adjacent non-cancerous tissues in formalin-fixed and paraffin-embedded tissue slides. The protein expression was comparably less in cancer tissues. The remaining GNAO1 staining in cancer cells mostly appeared close to the inner side of the cell membrane (Figure 1B and 1C).

### Down-regulation of GNAO1 increases the proliferation of HCC cells

**Figure 2:**
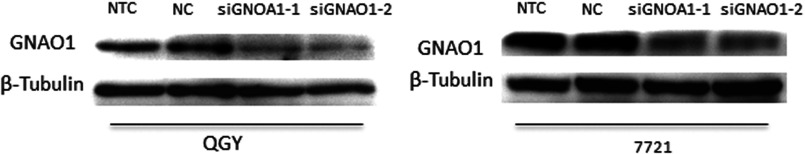
The inhibitory effect of GNAO1 gene expression by siRNAs in two human liver cancer cell lines: QGY-7703 and SMMC-7721 The cells were transfected with scrambled siRNA (NC) or without siRNA against human GNAO1. The protein level of GNAO1 in these cells 3 days after transfection was determined by Western blot analysis. Compared with scramble RNA, siGNAO1-1 and siGNAO1-2 were shown to remarkably suppress GNAO1 expression at the protein level.

**Figure 3:**
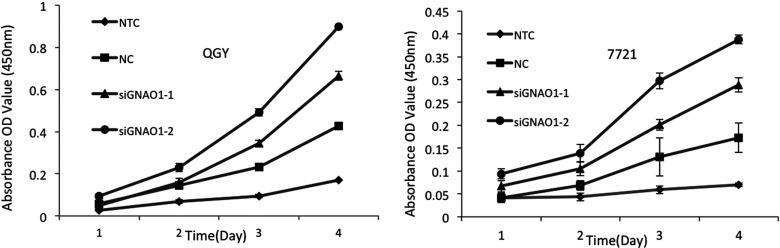
Cell proliferation test of QGY-7703 (upper panel) and SMMC-7721 (lower panel) after transfection with GNAO1 siRNAs (siGNAO1-1 and siGNAO1-2), scrambled siRNA (NC) or without transfection (NTC) The cell proliferation was determined by CCK-8 and expressed by absorbance. Cells transfected with GNAO1 siRNA were apparently grown faster than control groups, especially 48 h after transfection.

To observe the effect of GNAO1 on the HCC cells, two GNAO1 siRNA sequences were designed and transfected into QGY and 7721 cells. Through Western blot analysis (Figure 2), siGNAO1-1 and siGNAO1-2 both showed apparent suppression effect on GNAO1 and were used for the following studies. The cell proliferation was evaluated by CCK-8 assay (Figure 3). In the 96 h observation, we found that after 72 h of transfection, the cell proliferation was comparably faster in cells transfected with siGNAO1-1 and siGNAO1-2, in which the protein expression of GNAO1 was remarkably inhibited, than that in cells transfected with NC (*P*<0.05).

### Down-regulation of GNAO1 suppresses the senescence of HCC cells

**Figure 4:**
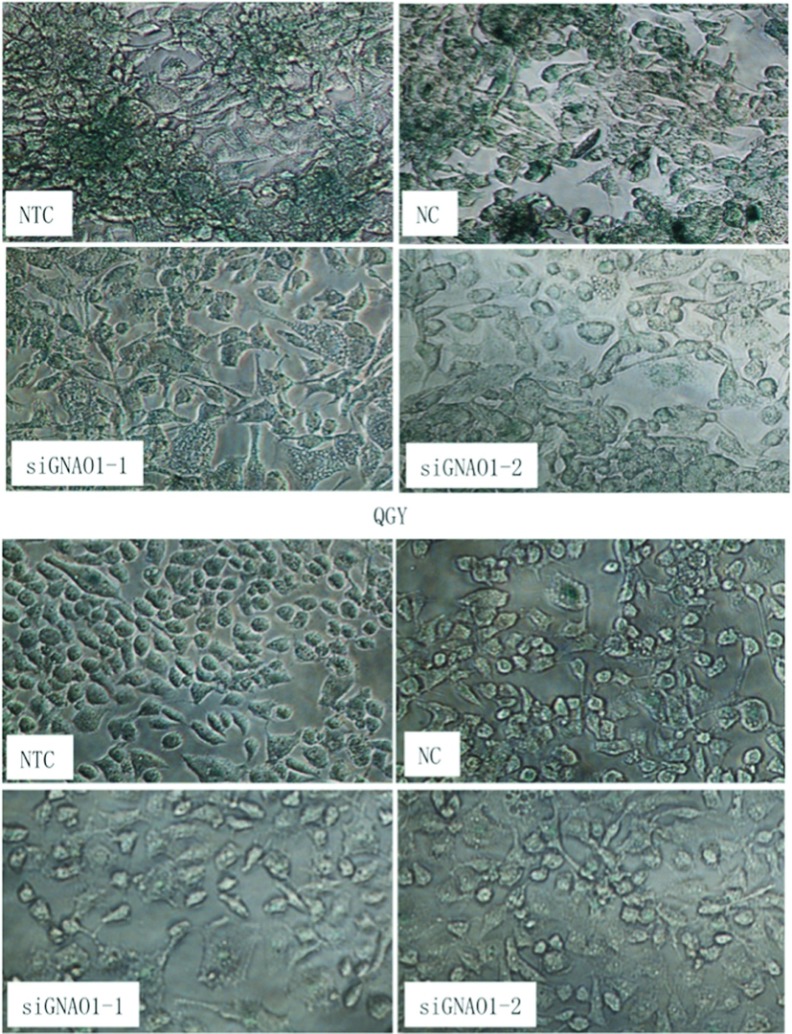
Cell senescence was stained by β-gal staining after transfection with NC, GNAO1 siRNA (siGNAO1-1 or siGNAO1-2) or without transfection Senescence in both QGY-7703 (upper panel) and SMMC-7721 (lower panel) were inhibited by siRNA transfection when compared with those transfected with scrambled siRNA.

Cell senescence is described as irreversible cell cycle arrest. Aside from growth arrest, senescent cells demonstrate characteristic metabolic changes, such as elevated SA-β-gal [17]. In β-gal staining assay, after 72 h of transfection, a lower proportion of senescent cells was observed in the group transfected with GNAO1 siRNA compared with that in the group of NC and NTC (Figure 4), indicating that down-regulation of GNAO1 inhibited the senescence of HCC cells.

## DISCUSSION

In the study of Jia et al. [16], the mRNA level of GNAO1 was also evaluated in tissues from HCC patients. In their study, all data was grouped into cancer and non-cancer and the difference was compared between these two groups. Whether the difference held in the paired tissues of each subject was not known. In our study, the difference was evaluated in each subject and we found that it held in almost all the subjects.

In this study, we have demonstrated a potential anticancer effect of GNAO1 on HCC and also provided data to suggest its possible mechanism. First of all, the relative expression of GNAO1 in HCC and adjacent non-cancerous tissues was estimated using real-time qPCR and IHC. Expectedly, the results of the two experiments were consistent, which demonstrated that the expression of GNAO1 was comparably less in HCC. Furthermore, in immunochemistry of HCC and adjacent non-cancerous tissue slices, we investigate the expressional position of GNAO1 protein. It was observed that GNAO1 proteins mostly appeared close to the inner side of the cell membrane.

To further investigate the mechanisms, we examined the function of GNAO1 by cell proliferation and cell senescence tests. Our data demonstrated that, importantly, the cell proliferation was comparably faster resulting from the absence of GNAO1, which manifested that GNAO1 was able to reduce cell growth. We also found that, when the cells were transfected with GNAO1 siRNA, the percentage of senescent cells was comparably less than that in the NC group. The effects of siRNA mediated knockdown of GNAO1 were not all-or-none, which was partially attributed to the insufficient transfection efficiency. However, as only one gene is altered, magnificent changes should not be expected. All these results supported that GNAO1 played a crucial role in the pathogenesis of HCC. It was strongly suggested that GNAO1 functioned as a negative regulator or tumour suppressor in HCC and the mechanism of its anticancer effect involved the inhibition of cell growth and the induction of cell senescence. Our findings provided a better understanding of the role of GNAO1 in the occurrence and development of HCC, which indicated that it was appropriate to be a new candidate biomarker as well as a potential therapeutic target of HCC.

To develop the clinical use of GNAO1, further studies may be required to investigate its role in cell signalling pathway. As a part of G protein, GNAO1 may have relations with OPRM1 and FZD2, which are G protein related upstream receptor and downstream effective module, respectively. OPRM1 gene encodes the mu-opioid receptor, which is a member of the opioid family of GPCRs (G-protein-coupled receptors) [18] and affects calcium current and potassium ion conductance [19,20]. FZD2 is a receptor of Wnt receptor pathway, which seems to be involved in the interactions with G-proteins [21,22].

In conclusion, we analysed the expression of GNAO1 in HCC and found that GNAO1 was down-regulated in cancerous tissues. We also investigated the value of the down-regulation of GNAO1 in HCC and revealed that it promoted cell proliferation and inhibited cell senescence. Indicatively, our findings offered a potential driving role in hepatic carcinogenesis, which suggested that GNAO1 could be a new biomarker as well as a promising therapeutic target for HCC. Our findings may facilitate the understanding of the molecular mechanisms of HCC.
